# Compound 48/80, a mast cell degranulator, causes oxidative damage by enhancing vitamin C synthesis via reduced glutathione depletion and lipid peroxidation through neutrophil infiltration in rat livers

**DOI:** 10.3164/jcbn.16-89

**Published:** 2017-04-20

**Authors:** Yosihiji Ohta, Koji Yashiro, Koji Ohashi, Yosuke Horikoshi, Chiaki Kusumoto, Tatsuya Matsura

**Affiliations:** 1 Department of Chemistry, Fujita Health University School of Medicine, 1-98 Dengakugakubo, Kutsukake-cho, Toyoake, Aichi 470-1192, Japan; 2 Department of Clinical Biochemistry, Faculty of Medical Chemistry, Fujita Health University School of Health Sciences, 1-98 Dengakugakubo, Kutsukake-cho, Toyoake, Aichi 470-1192, Japan; 3 Division of Medical Biochemistry, Department of Pathophysiological and Therapeutic Science, Totorri University Faculty of Medicine, 86 Nishimachi, Yonago, Tottori 683-8503, Japan; 4 Department of Gastroenterology, Nippon Kokan Fukuyama Hospital, 1844 Tsunoshita, Daimon, Fukuyama, Hiroshima 721-0927, Japan

**Keywords:** compound 48/80, mast cell degranulation, oxidative damage, neutrophil infiltration, rat liver

## Abstract

In this study, we examined whether compound 48/80 (C48/80), a mast cell degranulator, causes hepatic oxidative damage in rats. Serum and liver biochemical parameters were determined 0.5, 3 or 6 h after a single treatment with C48/80 (0.75 mg/kg). Serum histamine and serotonin levels increased 0.5 h after C48/80 treatment but diminished thereafter. Increases in serum vitamin C (VC) and transaminases and hepatic hydrogen peroxide, lipid peroxide, and myeloperoxidase levels and a decrease in hepatic reduced glutathione level occurred 0.5 h after C48/80 treatment and further proceeded at 3 h, but these changes diminished at 6 h. Serum lipid peroxide and hepatic VC levels increased 3 h after C48/80 treatment. Hepatic glycogen level decreased 0.5 h after C48/80 treatment and further decreased at 3 h. Pre-administered ketotifen diminished all these changes found at 3 h after treatment, while pre-administered NPC 14686 diminished these changes except changes in serum histamine and serotonin levels. Hepatocellular apoptosis observed at 3 h after C48/80 treatment was attenuated by pre-administered ketotifen and NPC 14686. These results indicate that C48/80 causes oxidative damage by enhancing VC synthesis via reduced glutathione depletion-dependent glycogenolysis and lipid peroxidation through neutrophil infiltration following mast cell degranulation in rat livers.

## Introduction

Compound 48/80 (C48/80) is a condensation product of *N*-methyl-*p*-methoxy phenylethylamine and formalin.^(1)^ This compound has been used to induce anaphylaxis and inflammation in experimental animals. It has been demonstrated that when rats are treated with C48/80, connective mast cells such as peritoneal mast cells are degranulated, resulting in release of mediators such as histamine and serotonin from the connective mast cells.^(2,3)^ We have reported that a single treatment of fasted rats with C48/80 causes a decrease in the level of reduced ascorbic acid, or vitamin C (VC), in the gastric mucosa at 3 h after treatment and in the adrenal gland at 0.5 and 3 h after treatment, while the C48/80 treatment causes an increase in VC level in the liver at 3 h after treatment.^(4–6)^ However, the mechanism by which a single C48/80 treatment causes an increase in hepatic VC level in rats is still unclear.

In the liver of mammals except primates including humans and guinea pigs, VC is synthesized via the uronic acid pathway, starting from glucose 6-phosphate.^(7)^ It has been reported that VC synthesis is stimulated by enhanced glycogenolysis in isolated mouse hepatocytes.^(8)^ It has been shown in rats treated once with C48/80 that hepatic glycogen is depleted by enhanced glycogenolysis, resulting in an increase in blood glucose level.^(9)^ It has also been shown in rats treated once with C48/80 that C48/80-induced glycogenolysis in the liver is independent of the content of glycogen stored in the tissue.^(10)^ Furthermore, it has been shown that degranulation of mast cells existing in the liver tissue of rats treated once with C48/80 is not associated with glycogen depletion in the tissue.^(11)^ Braun *et al.*^(12)^ have reported that depletion of reduced glutathione (GSH) induces glycogenolysis, resulting in stimulated VC synthesis in isolated mouse hepatocytes. Chan *et al.*^(13)^ have shown that glycogenolysis is involved in VC synthesis accompanied with GSH consumption in isolated rat hepatocytes. Bánhegyi *et al.*^(14)^ have shown that GSH is consumed during VC synthesis in isolated mouse hepatocytes and that hydrogen peroxide (H_2_O_2_), one of reactive oxygen species (ROS), is generated during VC synthesis accompanied with GSH consumption in the hepatocytes. These findings may allow us to assume that a single treatment of rats with C48/80 causes oxidative stress through VC synthesis via GSH depletion-dependent glycogenolysis in the liver tissue regardless of mast cell degranulation in the tissue.

It has been reported that hepatic blood flow shows an ischemia-reperfusion-like change by disruption of microcirculation in rats treated once with C48/80.^(15)^ It is known that ischemia-reperfusion due to disturbance of blood flow induces oxidative stress associated with ROS and lipid peroxidation in liver tissues.^(16)^ It is also known that neutrophil infiltration induces liver cell injury due to oxidative stress during ischemia-reperfusion.^(17)^ It has been shown that C48/80 treatment induces neutrophil infiltration into the liver of rats through mast cell degranulation *in vivo*.^(18)^

In the present study, therefore, we examined whether a single C48/80 treatment causes oxidative damage in the liver of rats by enhancing VC synthesis via GSH depletion-dependent glycogenolysis, H_2_O_2_ generation, lipid peroxidation, and neutrophil infiltration in the liver tissue through mast cell degranulation.

## Materials and Methods

### Chemicals

C48/80, ketotifen (KET), 3,3',5,5'-tetramethylbenzidine (TMB), methyl serotonin, and amyloglucosidase were purchased from Sigma-Aldrich Japan (Tokyo, Japan); NPC 14686 (NPC) from Funakoshi Co. (Tokyo, Japan); NADP^+^, ATP, glucose 6-phosphate dehydrogenase, and hexokinase from Oriental Yeast Co. (Tokyo, Japan); l-ascorbic acid (reduced form), *RRR*-α-tocopherol (Toc) and *RRR*-δ-toc which were used as the authentic standard and the external standard, respectively, glycogen, 2,2'-dipyridyl, 2-thiobarbituric acid (TBA), GSH, ethylendiaminetetraacetic acid (EDTA), gum Arabic, 5,5'-dithiobis(2-nitrobenzoic acid) (DTNB), *o*-phthalaldehyde, tetramethoxypropane, trichloroacetic acid (TCA), and other chemicals from Wako Pure Chem. Ind. (Osaka, Japan). All chemicals were used without further purification.

### Animals

Male Wistar rats aged six weeks were purchased from Nippon SLC Co. (Hamamatsu, Japan). The animals were housed in cages in a ventilated animal room with controlled temperature (23 ± 3°C) and relative humidity (55 ± 5%) with 12 h of light (7:00–19:00). The animals were maintained with free access to rat chow, Oriental MF (Oriental Yeast Co., Tokyo, Japan) and tap water for one week. All animals were maintained with free access to water and without food during the experiment. All animals received humane care in compliance with the guidelines of the Management of Laboratory Animals in Fujita Health University. The animal experiment was approved by the Institutional Animal Care and Use Committee, and its approved protocol number was M14-03.

### C48/80 treatment and KET and NPC administration

C48/80 (0.75 mg/kg body weight), dissolved in 0.9% NaCl, was intraperitoneally injected to 7-week-old Wistar male rats fasted for 24 h under non-anesthesia as described in our previous reports.^(4–6,19–22)^ The control rats received an i.p. injection of an equal volume of 0.9% NaCl. KET, a mast cell stabilizer,^(23)^ dissolved in 0.9% NaCl, at a dose of 1 mg/kg body weight was intraperitoneally injected 0.5 h before C48/80 treatment. NPC, an inhibitor of neutrophil recruitment,^(24)^ suspended in 5% gum Arabic, at a dose of 100 mg/kg body weight was orally administered 0.5 h before C48/80 treatment. The doses of KET and NPC used were determined according to our previous reports.^(5,19–21)^ All C48/80-untreated control rats received vehicle used for the preparation of 48/80 solution just before C48/80 treatment. A half of untreated control rats received vehicle used for the preparation of KET solution and the remaining untreated control rats received vehicle for the preparation of NPC suspension at 0.5 h before C48/80 treatment.

### Sample collection and determinations of serum and hepatic biochemical parameters

C48/80-treated and untreated control rats were sacrificed under pentobarbital anesthesia at 0.5, 3 or 6 h after C48/80 treatment at which time blood was collected from the inferior vena cava. C48/80-treated rats pretreated with and without KT or NPC and untreated control rats were killed under pentobarbital anesthesia at 3 h after C48/80 treatment at which time blood was collected from the inferior vena cava. Serum was obtained from the collected blood by centrifugation. Immediately after sacrifice, livers were perfused with ice-cold 0.9% NaCl through the portal vein to remove residual blood in the tissue as much as possible, and then the perfused livers were isolated. The isolated livers were washed in ice-cold 0.9% NaCl, wiped with filter paper, weighed, and then frozen on dry ice. The serum and livers obtained were stored at −80°C until used.

For serum serotonin and histamine determinations, serum samples were deproteinized by adding perchloric acid at a final concentration of 3% and then centrifuged at 4°C for 10 min (10,000 × *g*). Serum serotonin was measured by the method of Shibata *et al.*^(25)^ using high-performance liquid chromatography with electrochemical detection except that 40 mM sodium dihydrogenphosphate used for the mobile phase was replaced by 0.1 M citric acid-0.1 M sodium acetate (0.7:1.0, v/v). Methyl serotonin was used as an internal standard. Serum histamine was measured by the methods of Lorenz *et al.*^(26)^ and Shore *et al.*^(27)^ Histamine was reacted with *o*-phthalaldehyde and the intensity of the resultant fluorescence was measured using a spectrophotometer (the excitation wavelength, 360 nm; the emission wavelength, 450 nm). Serum transaminases, i.e., alanine aminotransferase (ALT) and aspartate aminotransferase (AST), were assayed using a commercial kit of Transaminase CII-Test (Wako Pure Chem. Ind., Ltd.). Serum VC was determined by the 2,2'-dipyridyl method of Zannoni *et al.*^(28)^ as follows: 0.5 ml of serum was mixed with 0.5 ml of ice-cold 10% TCA and then the mixture was centrifuged at 4°C (10,000 × *g*, 20 min). An aliquot of the supernatant obtained after deproteinization was used for the assay of VC. VC in each sample was measured by the 2,2'-dipyridyl method. The concentration of serum VC was determined using the standard curve of authentic l-ascorbic acid. Serum LPO was assayed by the TBA method of Yagi^(29)^ using tetramethoxypropane as a standard. The concentration of serum LPO is expressed as that of malondialdehyde (MDA) equivalents.

Liver tissues were homogenized in 9 volumes of ice-cold 0.15 M KCl containing 1 mM EDTA to prepare 10% homogenate. The prepared liver homogenate was used for the assays of VC, GSH, vitamin E (VE), H_2_O_2_, and LPO. VC in the liver homogenate was assayed by the same method as used for serum VC assay using l-ascorbic acid as a standard. GSH in the liver homogenate was assayed by the DTNB method of Sedlak and Lindsay^(30)^ using GSH as a standard. VE in the liver homogenate was assayed by the high-performance liquid chromatographic method with electrochemical detection using δ-Toc as an external standard as described in our previous report.^(5)^ The amount of liver VE is expressed as that of α-Toc. H_2_O_2_ in the liver homogenate was fluorometrically measured using an OxiSelect^TM^ Hydrogen Peroxide/Peroxidase assay kit (STA-344) from Cell Biolabs, Inc. (San Diego, CA) according to the manufacture’s instruction. LPO in the liver homogenate was assayed by the TBA method of Ohkawa *et al.*^(31)^ using tetramethoxypropane as a standard except that 1 mM EDTA was added to the reaction mixture. The amount of liver LPO is expressed as that of MDA equivalents. Liver glycogen was determined by the method of Brodal and Gehrken^(32)^ as follows: a part of liver tissues (50 mg, wet weight) was transferred to a glass test tube and then 0.5 ml of 5.4 M KOH was poured into the test tube. The test tube covered with a glass boll was heated at 100°C in a dry heating bath for 20 min and cooled in ice. A solution in the test tube was titrated with HCl (2-0.05 M) until a pH of about 7. The volume of the solution in the test tube was made up to 5.0 ml with distilled water. This prepared sample (0.1 ml) and glycogen standards (0.1 ml each) were mixed with 0.3 ml of 0.01 M acetate buffer (pH 4.6) and 10 µl of a solution of amyloglucosidase (5 U/ml) in 0.01 M acetated buffer (pH 4.6). After incubation at a room temperature for 30 min, these mixtures were further mixed with 1.42 ml of a mixed reagent solution which was prepared by mixing 1.22 ml of 0.1 M Tris-HCl buffer (pH 7.9), 0.1 ml of a mixture of 10 mM MgCl_2_, 1 mM NADP^+^, hexokinase (0.5 U), and glucose 6-phosphate dehydrogenase (1 U), and 0.1 ml of 5 mM ATP. The resultant mixtures were incubated at room temperature for 30 min. The blank was made without amyloglucosidase. The absorbance of the standards was measured at 340 nm and the glycogen concentrations calculated using an extinction coefficient of 6.22 mM^−1^cm^−1^. The glycogen content of liver samples was estimated on the basis of the standards from their absorbance readings. Liver MPO activity was determined as follows: liver tissues were homogenized in 9 volumes of ice-cold 0.05 M Tri-HCl buffer (pH 7.4). After sonication on ice for 20 s using a Handy Sonic model UR-20P (Tomy Seiko Co., Tokyo, Japan), the homogenate was centrifuged at 4°C (10,000 × *g*, 20 min). The resultant supernatant was dialyzed against 100 volumes of the same buffer for 1 h using a microdialysis device (molecular weight cut off 3500; Bio-Tec International Inc., Belleuve, WA). The dialyzed supernatant was incubated at 60°C for 2 h to increase the recovery of MPO in liver tissues according to the method of Schierwagen *et al.*^(33)^ MPO activity in the heat-treated liver sample was assessed by measuring the H_2_O_2_-dependent oxidation of TMB (dissolved in dimethylsulfoxide) at 37°C according to the method of Suzuki *et al.*^(34)^ This TMB oxidation was measured spectrophotometrically at 650 nm. One unit (U) of this enzyme activity is expressed as the amount of enzyme causing a change in absorbance of 1.0 per min per mg protein at 650 nm. Protein in each liver sample was assayed using a commercial Rapid Protein Assay kit (Wako Pure Chem. Ind. Ltd.). Bovine serum albumin was used as a standard in this protein assay.

### Histological examination

Liver sections, 5 µm thick, obtained from the largest liver lobes of rats sacrificed at 0, 0.5, 3 or 6 h after C48/80 treatment were processed routinely and stained with hematoxylin-eosin (H-E). Apoptosis was demonstrated *in situ* by the terminal deoxynucleotidyl transferase dUTP nick and labeling (TUNEL) assay. The TUNEL assay was performed using an ApoTag Peroxidase In Situ Apoptosis Detection kit from Chemicon International Inc. (Billerica, MA). The TUNEL assay in liver sections obtained from the largest liver lobes of rats sacrificed just before or 3 h after C48/80 treatment was conducted according to the manufacture’s recommendation. The histological changes in liver cells and tissues were examined by an experienced pathologist blinded to the treatment using a light microscope.

### Statistical analysis

All results obtained are expressed as the mean ± SD. The statistical analyses of the results were performed using a computerized statistical package (StatView II; Abacus Concepts Inc., Barkley, CA). Each mean value was compared by one-way analysis of variance (ANOVA) and Fisher’s protected least significance (PLSD) for multiple comparisons as the post-hoc test. The significance level was set at *p*<0.05.

## Results

### Effect of C48/80 treatment on serum serotonin, histamine, VC, and LPO levels and ALT and AST activities in rats

**Fig. 1:**
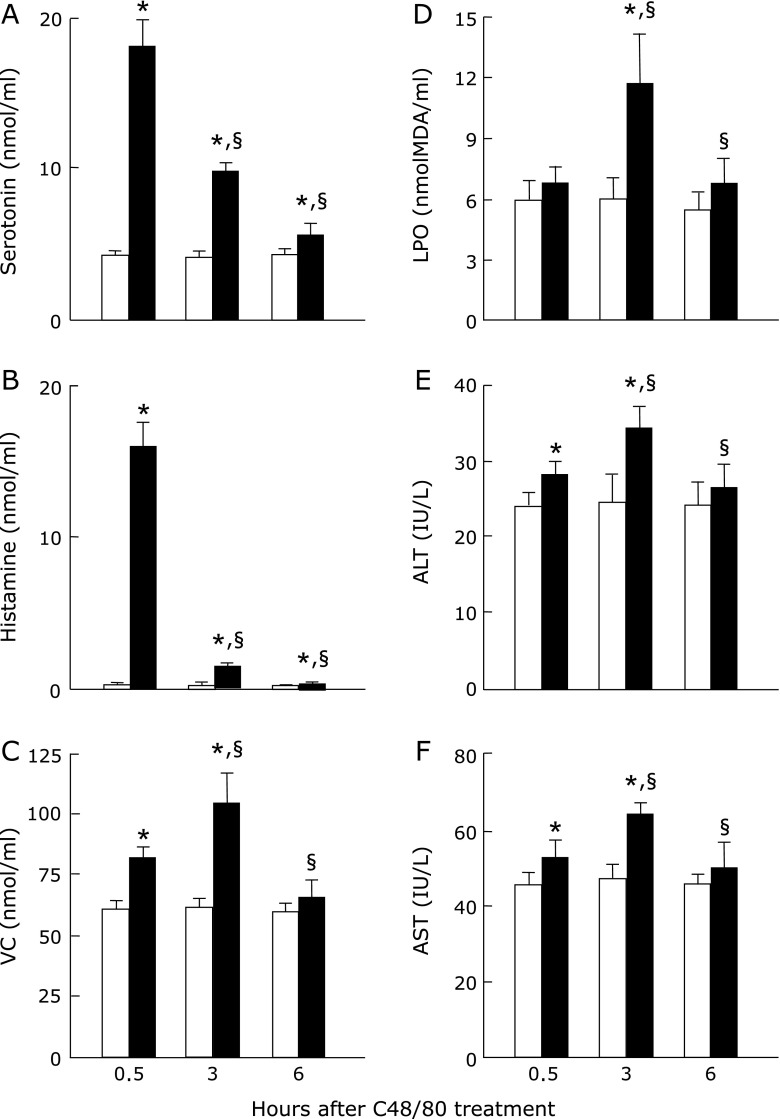
Time-related changes in serum serotonin, histamine, VC, and LPO concentrations and ALT and AST activities after a single C48/80 treatment. Serotonin (A), histamine (B), VC (C), LPO (D), ALT (E), and AST (F) were assayed in the serum of rats with and without a single treatment with C48/80 (0.75 mg/kg body weight, i.p.) at 0.5, 3 and 6 h after treatment as described in Materials and Methods. Open column, control rats; closed column, C48/80-treated rats. Each value is a mean ± SD (*n* = 5 for untreated control rats; *n* = 8 for C48/80-treated rats). *p<0.05 (vs control group); ^§^*p*<0.05 (vs the corresponding previous value).

Rats treated once with C48/80 showed significant increases in serum serotonin and histamine concentrations at 0.5, 3 and 6 h after treatment when compared with the corresponding untreated control rats (Fig. 1A and B). The increased serum serotonin and histamine concentrations at 0.5 h after C48/80 treatment dropped markedly at 3 h and further dropped to near the levels of untreated rats at 6 h (Fig. 1A and B). Serum VC concentration was significantly higher in C48/80-treated rats than in untreated control rats at 0.5 and 3 h after treatment but there was no significant difference in serum VC concentration between the treated and untreated groups at 6 h (Fig. 1C). Serum LPO concentration was significantly higher in C48/80-treated rats than in untreated control rats at 3 h after treatment but there were no significant differences in serum LPO concentrations at 0.5 and 6 h after treatment between the treated and untreated groups (Fig. 1D). Serum ALT and AST activities were significantly higher in C48/80-treated rats than in untreated control rats at 0.5 and 3 h after treatment but there were no significant differences in both enzyme activities between the treated and untreated groups at 6 h (Fig. 1E and F). The increases in serum VC concentration and ALT and AST activities in C48/80-treated rats were significantly larger at 3 h than at 0.5 h after treatment (Fig. 1C, E and F).

### Effect of C48/80 on liver histological changes in rats

**Fig. 2:**
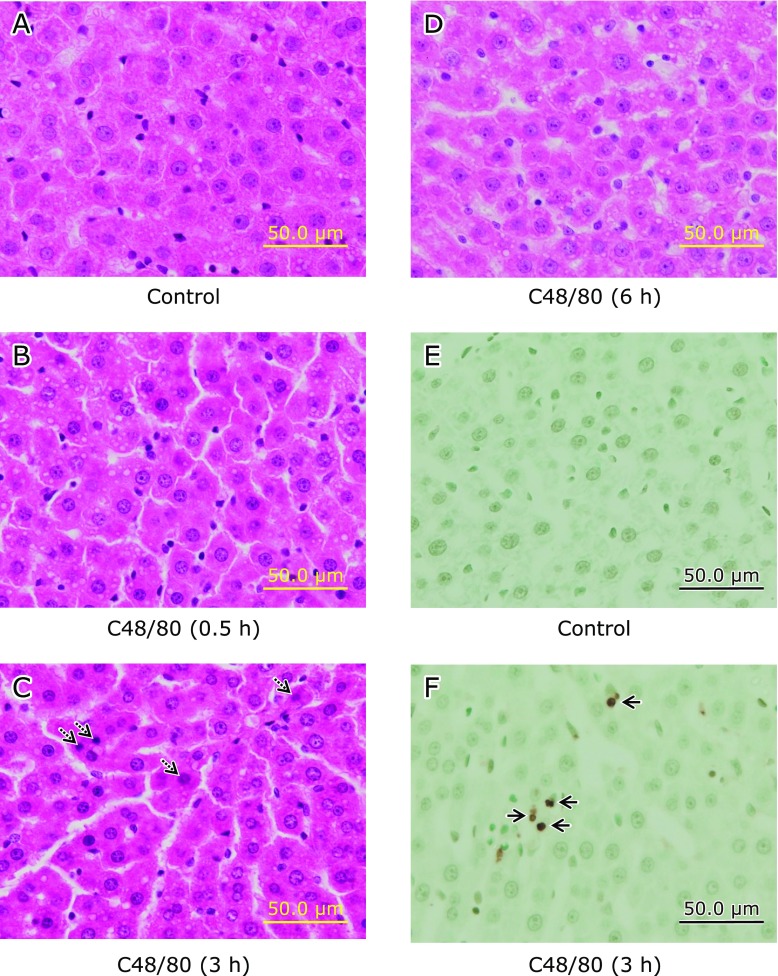
Time-related liver histological changes after a single C48/80 treatment. Liver histological changes were examined using H-E-stained (A–D) and TUNEL-stained (E and F) liver sections under a light microscope. Rats received a single treatment with C48/80 (0.75 mg/kg body weight, i.p.). (A) Liver section from untreated control rats sacrificed at 0.5 h after C48/80 treatment; (B) H-E-stained liver section from C48/80-treated rats (0.5 h after treatment); (C) H-E-stained liver section from C48/80-treated rats (3 h after treatment); (D) H-E-stained liver section from C48/80-treated rats (6 h after treatment); (E) TUNEL-stained liver section from untreated control rats sacrificed at 0.5 h after C48/80 treatment; (F) TUNEL-stained liver section from C48/80-treated rats (at 3 h after treatment). Dotted arrows indicate apoptotic bodies in liver cells; solid arrows indicate apoptotic liver cells (TNUEL-positive liver cells).

H-E-stained liver sections from C48/80-teated rats at 0.5, 3 and 6 h after treatment showed no apparent presence of necrosis and accumulated inflammatory cells in liver cells when compared with the H-E-stained liver section from untreated control rats (Fig. 2A–D). However, the presence of apoptotic bodies in liver cells was observed in the H-E-stained liver section from C48/80-teated rats at 3 h after treatment (Fig. 2C). There was no apparent apoptotic liver cell in the TUNEL-stained liver section from untreated control rats (Fig. 2E), while apoptotic liver cells (TUNEL-positive cells) were clearly observed in the TUNEL-stained liver section from C48/80-teated rats at 3 h after treatment (Fig. 2F).

### Effect of C48/80 treatment on hepatic VC, GSH, VE, and glycogen contents in rats

**Fig. 3:**
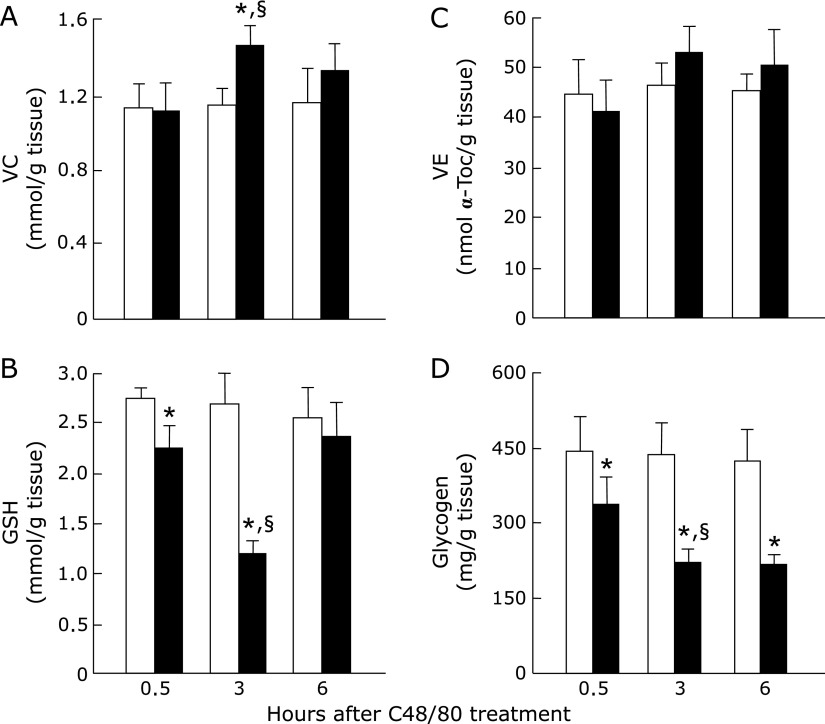
Time-related changes in liver VC, GSH, VE, and glycogen contents after a single C48/80 treatment. VC (A), GSH (B), VE (C), and glycogen (D) were assayed in the liver of rats with and without a single treatment with C48/80 (0.75 mg/kg body weight, i.p.) at 0.5, 3 and 6 h after treatment as described in Materials and Methods. Each value is a mean ± SD (*n* = 5 for untreated control rats; *n* = 8 for C48/80-treated rats). Open column, control rats; closed column, C48/80-treated rats. Each value is a mean ± SD (*n* = 5 for control rats; *n* = 8 for C48/80-treated rats). *p<0.05 (vs control group); ^§^*p*<0.05 (vs, the corresponding previous value).

C48/80-treated rats showed a significant increase in hepatic VC content at 3 h after treatment when compared with the corresponding untreated control rats but hepatic VC content in the treated group was not significantly different from that in the untreated group at 0.5 and 6 h (Fig. 3A). C48/80-treated rats showed a significant decrease in hepatic GSH content at 0.5 and 3 h after treatment when compared with the corresponding untreated control rats and the decrease in hepatic GSH content in the treated group was significantly larger at 3 h than at 0.5 h after treatment (Fig. 3B). At 6 h after C48/80 treatment, there was no significant difference in hepatic GSH content between treated and untreated rats (Fig. 3B). Hepatic VE content in C48/80-treated rats was not significantly different from that in untreated control rats at any time point after treatment (Fig. 3C). Hepatic glycogen content in C48/80-treated rats decreased significantly at 0.5, 3 and 6 h after treatment when compared with the corresponding untreated control rats and the decrease in hepatic glycogen content proceeded at 3 h without further proceeding at 6 h (Fig. 3D).

### Effect of C48/80 treatment on hepatic H_2_O_2_ and LPO contents, and MPO activity in rats

**Fig. 4:**
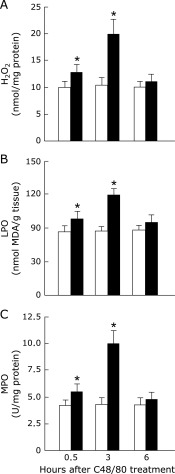
Time-related changes in liver H_2_O_2_ and LPO contents and MPO activity after a single C48/80 treatment. H_2_O_2_ (A), LPO (B), and MPO (C) were assayed in the liver of rats with and without a single treatment with C48/80 (0.75 mg/kg body weight, i.p.) at 0.5, 3 and 6 h after treatment as described in Materials and Methods. Each value is a mean ± SD (*n* = 5 for untreated control rats; *n* = 8 for C48/80-treated rats). Open column, control rats; closed column, C48-treated rats. Each value is a mean ± SD (*n* = 5 for control rats; *n* = 8 for C48/80-treated rats). *p<0.05 (vs control group); ^§^*p*<0.05 (vs the corresponding previous value).

C48/80-treated rats showed significant increases in hepatic H_2_O_2_ and LPO contents and MPO activity at 0.5 and 3 h after treatment when compared with the corresponding untreated control rats (Fig. 4A–C). The increases in hepatic H_2_O_2_ and LPO contents and MPO activity in the treated group were significantly larger at 3 h than at 0.5 h after treatment (Fig. 4A–C). At 6 h after C48/80 treatment, there were no significant differences in hepatic H_2_O_2_ and LPO contents and MPO activity between the treated and untreated groups (Fig. 4A–C).

### Effects of pre-administered KET and NPC on the levels of serum components and the activities of serum enzymes in rats treated with C48/80

**Fig. 5:**
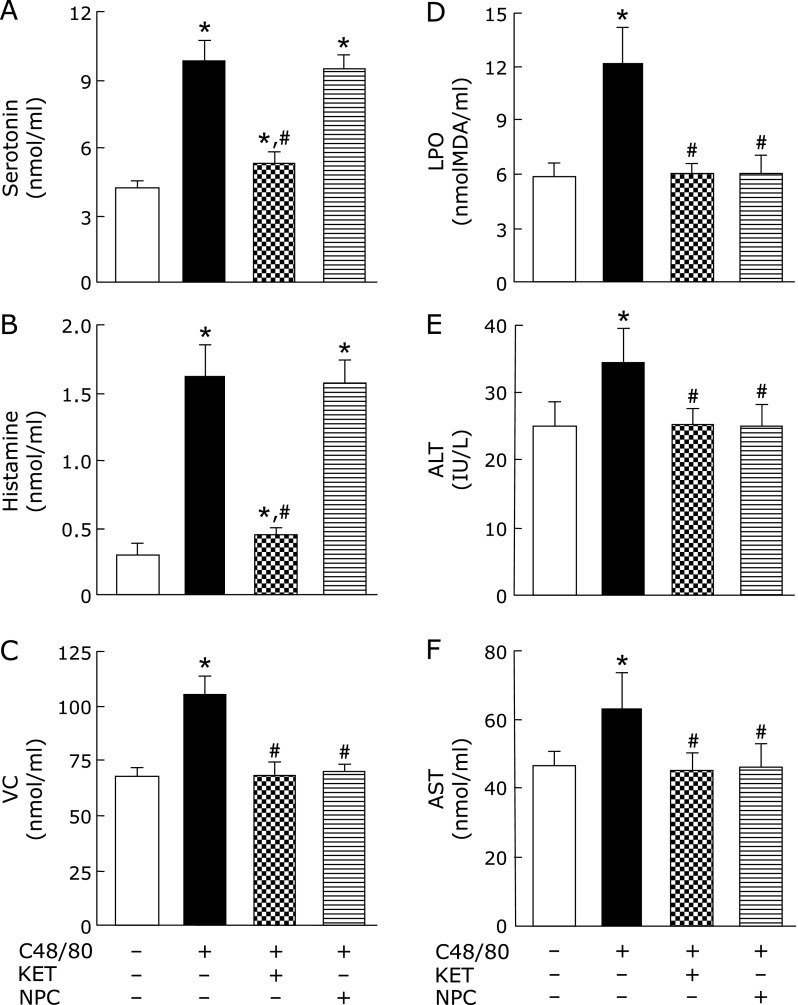
Effects of pre-administered KET and NPC on changes in serum serotonin, histamine, VC, and LPO concentrations and ALT and AST activities found at 3 h after C48/80 treatment. Rats received a single treatment with C48/80 (0.75 mg/kg body weight, i.p.). KET (1 mg/kg body weight, i.p.) or NPC (100 mg/kg body weight, p.o.) was administrated to C48/80-treated rats at 0.5 h before C48/80 treatment. All untreated control rats (*n* = 10) received vehicle used for the preparation of 48/80 solution just before C48/80 treatment. A half of untreated control rats (*n* = 5) received vehicle used for the preparation of KET solution and the remaining untreated control rats (*n* = 5) received vehicle for the preparation of NPC suspension at 0.5 h before C48/80 treatment. Serum serotonin (A), histamine (B), VC (C), and LPO (D), ALT (E), and AST (F) were assayed at 3 h after C48/80 treatment as described in Materials and Methods. Each value is a mean ± SD (*n* = 10 for untreated control rats; *n* = 8 for rats treated with C48/80 alone, C48/80-treated rats with KET pre-administration, and C48/80-treated rats with NPC pre-administration). *p<0.05 (vs control group); ^#^*p*<0.05 (vs group treated with C48/80 alone).

The increases in serum serotonin and histamine concentrations at 3 h after C48/80 treatment were significantly reduced by KET administered at 0.5 h before treatment (Fig. 5A). The serum serotonin and histamine concentrations in C48/80-treated rats with KET pre-administration were significantly, but slightly, higher than those in untreated control rats (Fig. 5A). NPC administered at 0.5 h before C48/80 treatment had no significant effect on the increases in serum serotonin and histamine concentrations at 3 h after treatment (Fig. 4B). The increases in VC and LPO concentrations and serum ALT and AST activities at 3 h after C48/80 treatment were significantly reduced by pre-administered KET and NPC (Fig. 5C–F). The serum VC and LPO concentrations and ALT and AST activities in C48/80-treated rats pre-administered with KET or NPC were not significantly different from those in untreated control rats (Fig. 5C–F).

### Effects of pre-administered KET and NPC on hepatocellular apoptosis in rats treated with C48/80

**Fig. 6:**
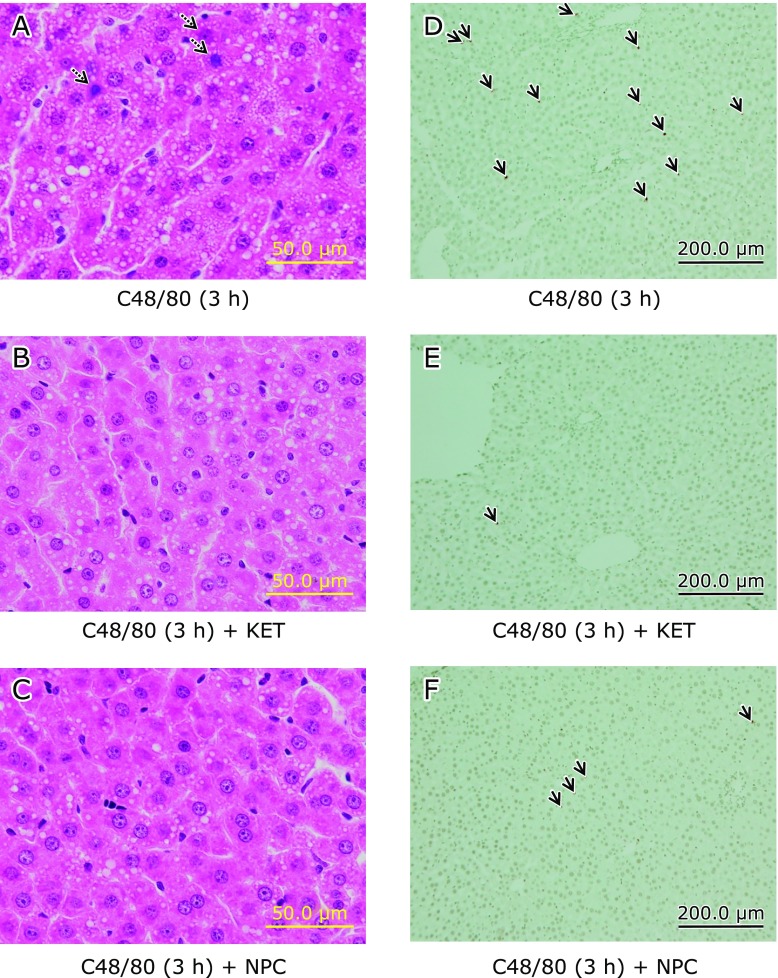
Effects of pre-administered KET and NPC on hepatocellular apoptosis in C48/80-treated rats. Rats received a single treatment with C48/80 (0.75 mg/kg body weight, i.p.). KET (1 mg/kg body weight, i.p.) or NPC (100 mg/kg body weight, p.o.) was administrated to C48/80-treated rats at 0.5 h before C48/80 treatment. Hepatocellular apoptosis was observed at 3 h after C48/80 treatment. (A) H-E-stained liver section from C48/80-treated rats; (B) H-E-stained liver section from C48/80-treated rats with KET pre-administration; (C) H-E-stained liver section from C48/80-treated rats with NPC pre-administration; (D) TUNEL-stained liver section from C48/80-treated rats; (E) TUNEL-stained liver section from C48/80-treated rats with KET pre-administration; (F) TUNEL-stained liver section from C48/80-treated rats with NPC pre-administration. Dotted arrows indicate apoptotic bodies in liver cells; solid arrows indicate apoptotic liver cells (TUNEL-positive liver cells).

In the H-E-stained liver section from C48/80-treated rats, there were apoptotic bodies in liver cells at 3 h after treatment, but the presence of apoptotic bodies in liver cells could not be found when KET or NPC was pre-administered to C48/80-treated rats (Fig. 6A–C). At the same time point, many apoptotic liver cells (TUNEL-positive cells) were observed in the TUNEL-strained liver section from C48/80-treated rats, but the number of apoptotic liver cells was apparently reduced in the TUNEL-strained liver section from C48/80-treated rats pre-administered with KET or NPC (Fig. 6D–F).

### Effects of pre-administered KET and NPC on hepatic VC. GSH, VE, and glycogen contents in rats treated with C48/80

**Fig. 7:**
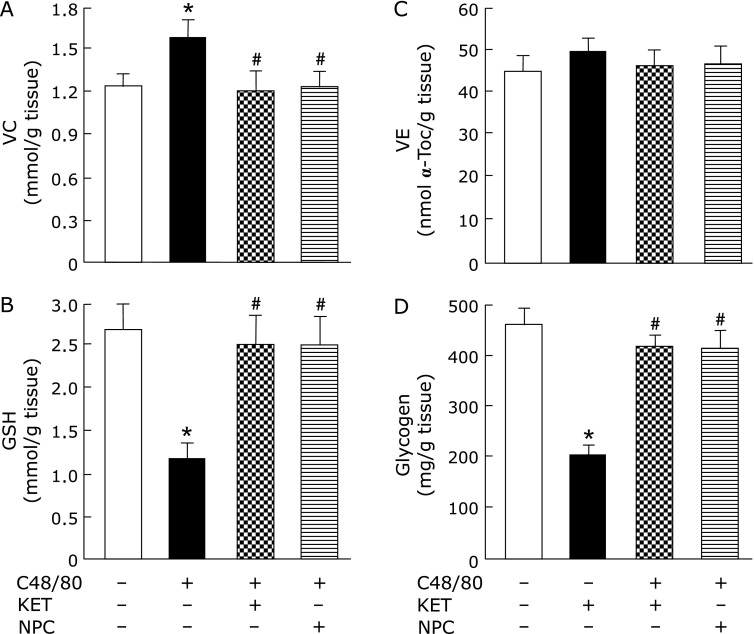
Effects of pre-administered KET and NPC on changes in liver VC, GSH, VE, and glycogen contents in C48/80-treated rats. Rats received a single treatment with C48/80 (0.75 mg/kg body weight, i.p.). KET (1 mg/kg body weight, i.p.) or NPC (100 mg/kg body weight, p.o.) was administrated to C48/80-treated rats at 0.5 h before C48/80 treatment. All untreated control rats (*n* = 10) received vehicle used for the preparation of 48/80 solution just before C48/80 treatment. A half of untreated control rats (*n* = 5) received vehicle used for the preparation of KET solution and the remaining untreated control rats (*n* = 5) received vehicle for the preparation of NPC suspension at 0.5 h before C48/80 treatment. Liver VC (A), GSH (B), VE (C), and glycogen (D) were assayed at 3 h after C48/80 treatment as described in Materials and Methods. Each value is a mean ± SD (*n* = 10 for untreated control rats; *n* = 8 for rats treated with C48/80 alone, C48/80-treated rats with KET pre-administration, and C48/80-treated rats with NPC pre-administration). *p<0.05 (vs control group); ^#^*p*<0.05 (vs group treated with C48/80 alone).

The increase in hepatic VC content and the decreases in hepatic GSH and glycogen contents at 3 h after C48/80 treatment were significantly attenuated by pre-administered KET or NPC (Fig. 7A, B and D). The hepatic VE content in C48/80-treated rats at 3 h after treatment was not significantly changed by pre-administered KET or NPC (Fig. 7C). In addition, the hepatic VC, GSH, and glycogen contents in C48/80-treated rats with KET or PNC pre-administration were not significantly different from those in untreated control rats (Fig. 7 A, B and D).

### Effects of pre-administered KET and NPC on hepatic H_2_O_2_ and LPO contents and MPO activity in rats treated with C48/80

**Fig. 8:**
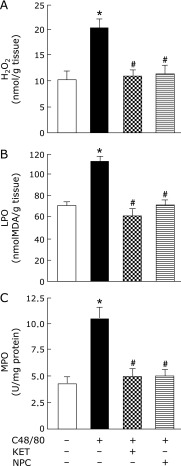
Effects of pre-administered KET and NPC on changes in liver H_2_O_2_ and LPO contents and MPO activity in C48/80-treated rats. Rats received a single treatment with C48/80 (0.75 mg/kg body weight, i.p.). KET (1 mg/kg body weight, i.p.) or NPC (100 mg/kg body weight, p.o.) was administrated to C48/80-treated rats at 0.5 h before C48/80 treatment. All untreated control rats (*n* = 10) received vehicle used for the preparation of 48/80 solution just before C48/80 treatment. A half of untreated control rats (*n* = 5) received vehicle used for the preparation of KET solution and the remaining untreated control rats (*n* = 5) received vehicle for the preparation of NPC suspension at 0.5 h before C48/80 treatment. Liver H_2_O_2_ (A), LPO (B), and MPO (C) were assayed at 3 h after C48/80 treatment as described in Materials and Methods. Each value is a mean ± SD (*n* = 10 for untreated control rats; *n* = 8 for rats treated with C48/80 alone, C48/80-treated rats with KET pre-administration, and C48/80-treated rats with NPC pre-administration). *p<0.05 (vs control group); ^#^*p*<0.05 (vs group treated with C48/80 alone).

The increases in hepatic H_2_O_2_ and LPO contents and MPO activity in C48/80-treated rats found at 3 h after treatment were significantly attenuated by pre-administered KET or NPC (Fig. 8 A–C). In addition, the hepatic H_2_O_2_ and LPO contents and MPO activity in C48/80-treated rats with KET or NPC pre-administration were not significantly different from those in untreated control rats (Fig. 8A–C).

## Discussion

In the present study, fasted rats treated once with C48/80 (0.75 mg/kg body weight, i.p.) showed apparent mast cell degranulation at 0.5 h after treatment, judging from the time-related changes in serum serotonin and histamine levels, as shown in our previous reports.^(5,19,21,22)^ In addition, C48/80-treated rats had apparent increases in the activities of serum transaminases, ALT and AST, indices of liver cell damage, at 0.5 and 3 h after treatment, but not at 6 h after treatment, although the increases in serum ALT and AST activities were higher at 3 h than at 0.5 h after treatment. In the H-E-stained liver section from C48/80-treated rats, however, no apparent presence of necrosis and accumulated inflammatory cells in liver cells could be observed at 0.5 and 3 h after treatment. Nevertheless, the presence of apoptotic bodies in liver cells was observed in the H-E-stained liver section from C48/80-treated rats at 3 h after treatment, indicating that C48/80 treatment causes hepatocellular apoptosis. The presence of hepatocellular apoptosis in C48/80-treated rats found at 3 h after treatment was confirmed by the observation of TUNEL-positive liver cells in the liver section. Thus, a single treatment of rats with 48/80 was found to induce cell damage in the liver tissue.

In the present study, rats treated once with C48/80 had a higher increase in serum VC concentration at 3 h than at 0.5 h after treatment but showed no increase in that concentration at 6 h. This time-related change in serum VC concentration after C48/80 treatment was well consistent with that shown in our previous reports.^(4–6)^ C48/80-treated rats also showed an increase in serum LPO concentration at 3 h after treatment as reported previously.^(5)^ Thus, oxidative stress occurred under the condition of increased serum VC level in rats treated once with C48/80. Rats treated once with C48/80 showed an increase in hepatic VC content at 3 h after treatment, as shown in our previous report.^(4)^ GSH exerts antioxidant action in a non-enzymatic manner and in an enzymatic manner via glutathione peroxidase under oxidative stress, resulting in its consumption.^(35,36)^ GSH is known to participate in recycling of VC from oxidized VC in an enzymatic manner and a non-enzymatic manner.^(5,37)^ In addition, it has been shown that VC synthesis is stimulated under GSH deficiency in the liver of mice.^(38)^ In the liver of rats treated once with C48/80, GSH content decreased 0.5 h after treatment and further decreased at 3 h, but the decreased GSH content was returned to the untreated control level at 6 h. Thus, a decrease in hepatic GSH content began before the occurrence of an increase in hepatic VC content in rats treated with C48/80. VE is known to exert antioxidant action through scavenging of ROS, especially singlet oxygen, by itself and by breaking the chain reaction of lipid peroxidation occurring in cell membranes.^(39)^ VE is recycled from VE radical generated under oxidative stress by VC, resulting in its efficient antioxidant action and its restoration.^(7,39,40)^ However, there was no change in hepatic VE content in rats treated once with C48/80. This unchanged VE content in the liver of C48/80-treated rats might be due to the recycling of VE by VC increasing in the tissue. It has been demonstrated that H_2_O_2_ is generated during VC synthesis accompanied with GSH depletion in isolated mouse hepatocytes.^(14)^ LPO is generated by peroxidation of unsaturated fatty acids present in cell membranes via ROS under oxidative stress.^(41)^ In rats treated once with C48/80, increases in hepatic H_2_O_2_ and LPO contents occurred 0.5 h after treatment followed by their further increases at 3 h, but these increases diminished completely at 6 h. Thus, H_2_O_2_ and LPO contents in the liver of C48/80-treated rats increased under GSH depletion accompanied with an increase in VC content in the tissue. It has been reported that when isolated rat hepatocytes with GSH depletion induced by 1-bromoheptane or phorone treatment are treated with cumene hydroperoxide as an oxidative stressor, VC synthesis is stimulated in the cells, resulting in an increase in VC content in the cells.^(42)^ Therefore, one can suggest that the increase in VC content in the liver of C48/80-treated rats could be related to oxidative damage associated with GSH depletion followed by H_2_O_2_ generation and lipid peroxidation. It has been shown that GSH depletion-dependent glycogenolysis occurs in isolated mouse and rat hepatocytes.^(8,12)^ In the present study, fasted rats treated once with C48/80 had decreased hepatic glycogen content at 0.5 h after treatment and further decreased hepatic glycogen content at 3 and 6 h, although the decreased hepatic glycogen content was almost equal at 3 and 6 h. It is known that a single C48/80 treatment enhances glycogenolysis in the liver of rats, resulting in glycogen depletion in the tissue.^(9)^ It is also known that enhanced glycogenolysis in the liver of rats treated once with C48/80 is independent of the content of glycogen stored in the tissue.^(10)^ Therefore, these findings and the above-described results allow us to suggest that, in the liver of rats with a single C48/80 treatment, H_2_O_2_ generation and lipid peroxidation are enhanced following VC synthesis via GSH depletion-dependent glycogenolysis, resulting in the occurrence of oxidative damage.

It has been shown that an ischemia-reperfusion-like change in hepatic blood flow through disruption of microcirculation in rats with a single intravenous injection of C48/80 is prevented by pre- or simultaneous administration of lodoxamide, an inhibitor of mast cell degranulation.^(15)^ It has also been shown that C48/80 treatment induces neutrophil infiltration into the liver tissue of rats through mast cell degranulation *in vivo*.^(18)^ It is known that liver injury is caused by oxidative stress due to generated ROS and enhanced lipid peroxidation during ischemia-reperfusion induced by disturbance of hepatic blood flow.^(16)^ It has been demonstrated that infiltrated neutrophils induce oxidative stress due to ROS such as O_2_^•−^ and H_2_O_2_ generated by NADPH-oxidase and hypochlorous acid generated by MPO in the presence of H_2_O_2_ and chloride ion in liver tissues under the condition of ischemia-reperfusion.^(17)^ As to the mechanism for C48/80-induced neutrophil infiltration into the liver tissue of rats through mast cell degranulation, Reilly *et al.*^(18)^ have suggested that C48/80-induced neutrophil adherence to the endothelium of sinusoid and central and sublobular venules in the liver of rats through mast cell degranulation is mediated by serotonin and other mast cell constituents (e.g., prostaglandins, platelet-activating factor, and heparin), but at sites other than histamine and serotonin-specific receptors. It has also been shown that a single C48/80 treatment causes neutrophil accumulation in the gastric mucosa and skeletal muscle of rats through mast cell degradation.^(19,20,43)^ In the present study, a single treatment of rats with C48/80 increased MPO activity, a marker of tissue neutrophil infiltration, in the liver tissue at 0.5 h after treatment and further increased in the tissue at 3 h, but the increase in that activity diminished completely at 6 h. Thus, C48/80 treatment was found to induce neutrophil infiltration into the liver tissue of rats. It is known that activated neutrophils mediate lipid peroxidation in liposomes via the respiratory burst *in vitro*.^(44)^ It is also known that MPO induces lipid peroxidation in liposomes in the presence of H_2_O_2_ and chloride ion *in vitro*.^(45)^ Besides, it is known that GSH is oxidized in the system of MPO, H_2_O_2_, and a halide ion *in vitro*.^(46)^ Accordingly, these findings allow us to assume that an enhancement of lipid peroxidation in the liver of rats treated once with C48/80 is, at least in part, mediated by neutrophils infiltrating into the tissue and that infiltrated neutrophils contribute to the occurrence of oxidative damage by causing GSH depletion, ROS generation, and lipid peroxidation in the liver of C48/80-treated rats. However, a possibility that ROS generated in activated mast cells contribute to oxidative damage in the liver of C48/80-treated rats cannot be ruled out, because it is known that ROS generation occurs with histamine release in rat peritoneal mast cells treated with C48/80.^(47)^

Next, we attempted to clarify whether a single C48/80 treatment causes oxidative damage in the liver of rats through mast cell degranulation. Administration of KET, a mast cell stabilizer,^(23)^ to C48/80-treated rats at 0.5 h before treatment diminished not only the increases in serum serotonin and histamine levels but also the increases in serum VC and LPO levels and ALT and AST activities found at 3 h after treatment almost completely. The histological observation in H-E-stained and TUNEL-stained liver sections showed that hepatocellular apoptosis in C48/80-treated rats found at 3 h after treatment was apparently diminished by KET pre-administration. In addition, KET pre-administered to C48/80-treated rats diminished the increases in hepatic VC, H_2_O_2_, and LPO contents and MPO activity and the decreases in hepatic GSH and glycogen contents found at 3 h after treatment almost completely. As described above, it has been shown that mast cell degranulation occurs in the liver of rats treated once with C48/80, which induces an ischemia-reperfusion-like change in blood flow and neutrophil infiltration in the tissue.^(15,18)^ It has also been shown that degranulation of mast cells existing in the liver tissue of rats treated one with C48/80 is not associated with glycogen depletion in the tissue.^(8)^ Accordingly, talking these findings into consideration, the above-described results indicate that oxidative damage in the liver of rats treated once with C48/80 occurs through degranulation of mast cells existing, perhaps, not only in the hepatic tissue, but also in the extrahepatic tissues.

Furthermore, we attempted to clarify whether hepatic damage in rats treated once with C48/80 is related to oxidative stress occurring through the infiltration of neutrophils into the liver tissue. Administration of NPC, an inhibitor of neutrophil recruitment,^(24)^ to C48/80-treated rats at 0.5 h before treatment attenuated the increased serum VC, H_2_O_2_, and LPO levels and ALT and AST activities found at 3 h after treatment to near the levels of C48/80-untreated rats without affecting the increases in serum serotonin and histamine levels. The histological observation in H-E-stained and TUNEL-stained liver sections showed that NPC pre-administration attenuated the occurrence of hepatocellular apoptosis in C48/80-treated rats found at 3 h after treatment. In addition, the pre-administration of NPC to C48/80-treated rats reversed the changed hepatic VC, GSH, H_2_O_2_, LPO, and glycogen contents and MPO activity found at 3 h after treatment to near the levels of C48/80-untreated rats. Taking the above-described time-related changes in liver VC, GSH, H_2_O_2_, LPO, and glycogen levels and MPO activity into consideration, these results allow us to indicate that VC synthesis via GSH depletion-dependent glycogenolysis, which results in H_2_O_2_ generation, and lipid peroxidation in the liver of rats treated once with C48/80 occur due to neutrophils infiltrating into the tissue, resulting in the occurrence of oxidative damage associated with GSH depletion, H_2_O_2_ generation, and lipid peroxidation in the tissue.

In conclusion, the results obtained in the present study indicate that a single treatment of rats with C48/80 causes oxidative damage by enhancing VC synthesis via GSH depletion-dependent glycogenolysis, which results in H_2_O_2_ generation, and lipid peroxidation through neutrophil infiltration following degranulation of mast cells existing, perhaps, in the hepatic and extrahepatic tissues. However, further investigations are needed to clarify the source of degranulated mast cells involved in C48/80-induced hepatic oxidative damage and how mediators such as histamine and serotonin released from degranulated mast cells contribute to C48/80-induced hepatic oxidative damage.

## Abbreviations

ALT: alanine aminotransferase
AST: aspartate aminotransferase
C48/80: compound 48/80
DTNB: 5,5'-dithiobis(2-nitrobenzoic acid)
EDTA: ethylenediaminetetraacetic acid
GSH: reduced glutathione
H-E: hematoxylin-eosin
KET: ketotifen
LPO: lipid peroxide
MDA: malondialdehyde
MPO: myeloperoxidase
NPC: NPC 14686
TBA: 2-thiobarbituric acid
TCA: trichloroacetic acid
TMB: 3,3',5,5'-tetramethylbenzidine
Toc: tocopherol
TUNEL: terminal deoxynucleotidyl transferase dUTP nick and labeling
VC: vitamin C
VE: vitamin E
